# Geometric Effect for Biological Reactors and Biological Fluids

**DOI:** 10.3390/bioengineering5040110

**Published:** 2018-12-13

**Authors:** Kazusa Beppu, Ziane Izri, Yusuke T. Maeda, Ryota Sakamoto

**Affiliations:** Department of Physics, Kyushu University, Fukuoka 819-0395, Japan; kazu.beppu@phys.kyushu-u.ac.jp (K.B.); ziane.izri@phys.kyushu-u.ac.jp (Z.I.); sakaryo@phys.kyushu-u.ac.jp (R.S.)

**Keywords:** geometry, synthetic biology, cell-free system, collective dynamics

## Abstract

As expressed “God made the bulk; the surface was invented by the devil” by W. Pauli, the surface has remarkable properties because broken symmetry in surface alters the material properties. In biological systems, the smallest functional and structural unit, which has a functional bulk space enclosed by a thin interface, is a cell. Cells contain inner cytosolic soup in which genetic information stored in DNA can be expressed through transcription (TX) and translation (TL). The exploration of cell-sized confinement has been recently investigated by using micron-scale droplets and microfluidic devices. In the first part of this review article, we describe recent developments of cell-free bioreactors where bacterial TX-TL machinery and DNA are encapsulated in these cell-sized compartments. Since synthetic biology and microfluidics meet toward the bottom-up assembly of cell-free bioreactors, the interplay between cellular geometry and TX-TL advances better control of biological structure and dynamics in vitro system. Furthermore, biological systems that show self-organization in confined space are not limited to a single cell, but are also involved in the collective behavior of motile cells, named active matter. In the second part, we describe recent studies where collectively ordered patterns of active matter, from bacterial suspensions to active cytoskeleton, are self-organized. Since geometry and topology are vital concepts to understand the ordered phase of active matter, a microfluidic device with designed compartments allows one to explore geometric principles behind self-organization across the molecular scale to cellular scale. Finally, we discuss the future perspectives of a microfluidic approach to explore the further understanding of biological systems from geometric and topological aspects.

## 1. Introduction

The surface plays significant roles to orchestrate various functions ranging from surface conduction in topological insulators [1,2,3] to surface-catalyzed chemical reactions [4,5]. In biology, the smallest functional and structural unit defined by the micro-scale compartment is the cell. Owing to the great surface to volume ratio of tiny cells, the membrane interface is not just a solid wall, but plays pivotal roles for internal bio-chemical reactions and cellular mechanics. Cell-size regulates its growth rate and progression of the cell cycle [6], and active cytoskeleton steers the cellular shape and its motility [7]. Thus, the roles of physical confinement on intracellular bio-chemical and mechanical activities will be presented in this review article.

Despite the significant advances in single-cell measurements, it is still challenging to elucidate the underlying mechanisms in biological phenomena, due to the inherent complexity of the cellular environment. Thus, it is necessary to have model platforms to isolate the important features of cells to find out fundamental principles in living systems. In this paper, we will describe the significant benefits of microfluidics by answering the two following questions:

1. How are such experimental platforms utilized for research on biological systems?
2. What have these technologies allowed us to understand?

We will discuss recent experimental advances in understanding the biological functions in vitro. We will further emphasize how these experimental studies have broad implications from cell-free gene expression to collective cell dynamics, and beyond.

## 2. The Geometric Effect for Cell-Free Bioreactors

### 2.1. Cell-Free Expression as a Live-Cell Mimic

The minimal gene set necessary for a cell to live, identified by reconstructing synthetic microbes, contains less than 500 genes [8]. However, it is yet difficult to elucidate the mechanisms at play in the accurate regulation of these gene networks, both in time and amount, because gene regulation and its interaction with the outer environment is still a vast parameter space to explore. To uncover those mechanisms, the bottom-up approach synthesizing simplified gene circuits, and running gene expression in vitro has proved itself a reliable tool.

Such achievement was made possible by the development of extra-cellular media capable of hosting gene expression. A simple way to achieve that is to extract cell lysate and perform gene expression in this medium. The work of Zubay et al. (1973) has served as a model protocol to produce a cell-free extract from various kinds of living cells [9]. Since then, that kind of system has been significantly improved in yield and versatility [10]. Although numerous commercial solutions have been developed, it is only recently that Shin et al. (2012) developed a cell-free system that could host protein synthesis in liposomes [11,12]. Such an extra-cellular medium gives high protein synthesis yield in addition to a high versatility on possible genes to express, but at the expense of the detailed knowledge of the metabolic processes at play. Finally, one can also completely reconstitute an extra-cellular medium from purified species. Each component of the gene expression machinery is present in a known amount. This is the PURE system developed by Shimizu et al. (2001) [13,14]. This system offers a completely controlled extra-cellular gene expression platform, however with a lower yield and versatility.

A cell-free transcription-translation system, such as the PURE system (using purified proteins) and TX-TL (using a cell-free extract from *Escherichia coli*) is an ideal platform to quantitatively characterize the relationship between genetic information encoded in DNA and the function of synthesized reporter proteins. These in vitro cell-free expression (CFE) systems enable us to perform prototyping of gene circuits [11], self-assembly of bacteriophage from its DNA [12], and mathematical modeling of simple reactions [15,16].

#### 2.1.1. Compartmentalized Gene Expression

Compartmentalization is one of the critical features of living systems. That is why the first challenge in mimicking living systems was to first control the confinement of biological material. The first artificial object encapsulating biological material was created about half a century ago [17]. At that time, there was no genetic information—no DNA—but only proteins in the cell-sized compartment. At the end of the 20th century, microtubules in a lipid vesicle could alter the shape of that vesicle, thus showing that biological functions can be reproduced in confined artificial systems [18]. It is only recently that the first artificial system was able to produce secondary metabolites, such as proteins, in liposomes, from bacterial DNA in vitro [11].

Two approaches are possible to understand the effect of confinement on gene expression. The top-down approach requires studying actual living systems and deriving their properties from the observation of grown populations of cells. In those conditions, it is very challenging to control their physical parameters at play accurately and one has to rely on stochasticity to explore the parameters space efficiently. On the contrary, the bottom-up approach, artificial and simplified, allows one to design and individually control the parameters deemed significant. With the help of microfluidics, one can easily fabricate an experimental setup that generates and enables the observation of thousands of individual micro-reactors. The later can take a wide variety of forms, including, but not limited to droplets, liposomes, and microwells.

**Figure 1:**
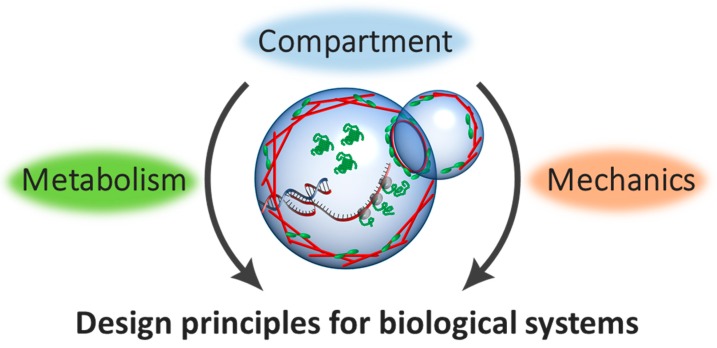
Basic components of the cell. The cell-sized compartment is comprising of cytoskeleton beneath the membrane (red rods, green motor-proteins), metabolism for molecules (green) synthesized by gene expression from DNA (red and blue double helix).

When we look at the cell, its functions are mainly divided into two components: Metabolism and mechanics (Figure 1). The metabolism includes gene expression, signaling, biochemical reactions, while the mechanics include cell migration, cell division, and mechano-sensing on the membrane. The genetic information encoded within DNA controls these functions. Therefore, the design of DNA to understand emergent metabolism and mechanics is the central theme of the Synthetic Biology field. To begin with, we focused on how the information propagates from DNA molecules to biological structure and dynamics, then move onto the mechanical nature of the cell. Finally, we briefly touch the recent progress on the communication between compartments, and how these two features are united as living cell-like manner toward next level complexity.

#### 2.1.2. Gene Expression in Liposome

**Figure 2:**
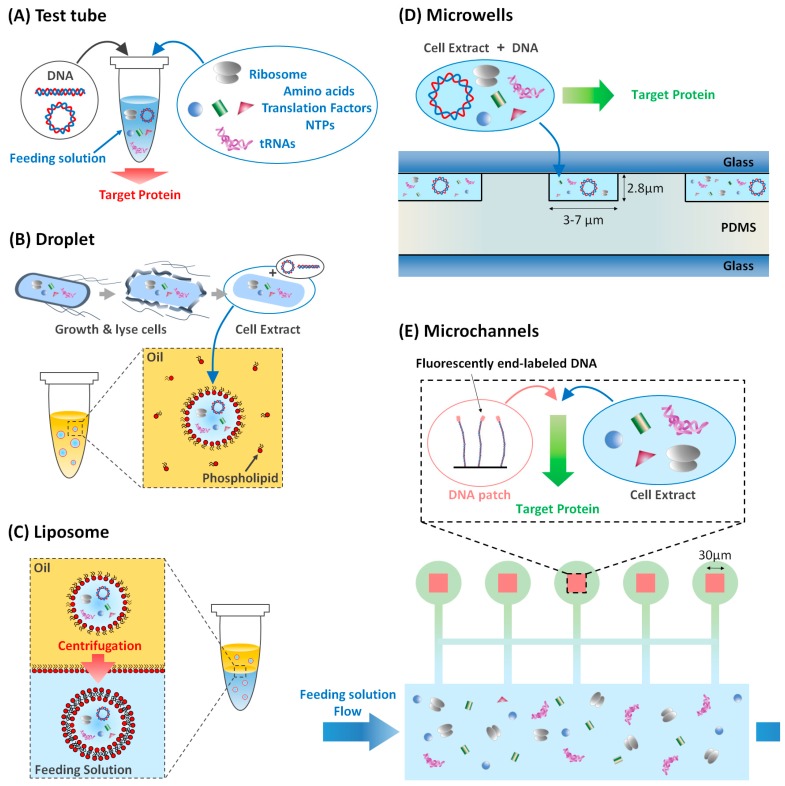
Cell-free TX-TL (transcription-translation) reactors in various compartments. (**A**) Test tube. Shimizu et al. have been developed the PURE system [13] in which purified transcription/translation factors, nutrients and DNA are incorporated into a test tube to produce target proteins. (**B**) Noireaux et al. have been developed TX-TL extract taken from bacteria *Escherichia coli* which contains TX-TL factors and nutrients to produce proteins [19]. PURE system and TX-TL extract can be encapsulated into a water-in-oil droplet, which recapitulates the micron-sized boundary of living cells [16]. (**C**) Liposome, which is covered with the lipid bilayer and surrounded by feeding solution, can be generated from the interface-transfer method by centrifugation through water-oil interface [19]. (**D**) Gasket design of a cylindrical cell-free genetic micro-reactor [21]. (**E**) Array of five coupled DNA compartments, communicating with a feeding solution as described in [22].

In conjunction with microfluidic technology, this bottom-up approach has much grown up for the last decades (Figure 2A). One of the simplest, but robust, examples is to encapsulate reaction system inside lipid bilayer vesicles to recapitulate gene expression in a single cell. Firstly, injecting a bulk solution of the CFE system into a lipid-oil mixture followed by a gentle mixing yields water-in-oil droplets covered with lipid monolayer (Figure 2B). Next, these water-in-oil droplets are transferred through the oil-water interface by centrifugation, making the liposome covered with lipid bilayer membrane as a cell-mimics (Figure 2C). Noireaux and Libchaber (2004) encapsulated DNA circuits encoding GFP (green fluorescent protein) as a reporter [19]. For several hours after encapsulation, the fluorescent intensity inside a droplet increases as time advances, meaning that the amount of synthesized GFP is increased by gene expression. GFP synthesis is just an event performed in bulk space within the droplet. The interesting feature of the cell is not just compartmentalization, but also the interaction with outer space through lipid bilayer membrane. Can one implement a function in the genetic code that interacts with other species? To address this challenge, they designed DNA encoding the genetic code of channel protein α-hemolysin, that creates membrane pores to exchange molecules in a droplet with nutrients in the external environment [19,20]. After running gene expression, channel protein builds membrane pores through the lipid bilayer, enabling the long-lasting gene expression for a week, beyond the few hours previously mentioned. The pure molecular function of α-hemolysin membrane channel is directly evaluated by synthesizing that molecule in the vesicle, in a quantitative manner.

To further realize interaction with outer environment beyond nutrients exchange, Majumder et al. (2017) developed cell-mimics capable of sensing their physical and chemical environment [23]. They prepared liposomes encapsulating DNA coding mechano-sensitive membrane protein MscL that senses an increase in membrane tension and opens a pore through the lipid bilayer. The liposome is prepared by ultra-thin double emulsion method by droplet microfluidics [24]. In short, by using glass capillary device, the flow of three different phases—outer solution, volatile oil, and cell lysate—are encapsulated inside the ultra-thin double emulsion. A lipid bilayer vesicle will form when all the oil evaporates. To couple mechanical input to biochemical sensing, they co-expressed G-GECO (calcium sensitive reporter) and MscL inside a liposome in feeding solution containing Ca^2+^ ions. Under the iso-osmotic state, there was no G-GECO fluorescence. In contrast, the hypo-osmotic condition of ~100 mOsm osmotic difference leads to G-GECO fluorescence, indicating that osmotic pressure gradient across the lipid bilayer membrane changes the membrane tension, thereby MscL channel opens. Consequently, Ca^2+^ ions entered into the liposome through the lipid membrane. Thus, microfluidics allows one to control osmotic pressure and investigate the biological functions from gene-expression, metabolism through the membrane, to mechanical input for bio-sensing.

The function of the membrane surface is not limited to nutrient exchange, but also works as a scaffold for active cytoskeleton. As for bacterial cytoskeleton, MreB filaments are responsible for maintaining the rod-shaped polarity [25]. Maeda et al. (2012) designed and encapsulated DNA encoding cytoskeleton proteins MreB into liposome, and MreB proteins were expressed and self-organized beneath the membrane into thick filaments with average contour length of 10 µm [26,27]. Remarkably, these thick filaments did not appear in in vitro bulk reactions, where the filaments are 100 times shorter than the ones formed in liposomes. These findings point out the importance of the cell-sized confinement to successfully functionalize specific proteins, where the interaction with the membrane surface is crucial for the self-organization of molecules.

#### 2.1.3. The Effect of the Spatial Confinement on Gene Expression

Spatial confinement is necessary to define a ‘cell’, firstly discovered and named by Robert Hooke in 1665 [28]. The size of actual living cells and reconstituted liposome shows variability, both across a given population and with time in a chosen individual. This might affect the mechanisms at play in gene expression. As of today, the link between cell size and its protein production is not clear. Indeed, one could expect that the larger the cell, the larger its genetic material. However, the scaling laws involved are not necessarily trivial; it is yet unclear how genome regulation is tuned to the size of the cell [29].

Although it is challenging to set the size of cells, one can create micro-reactors of a given volume and study gene expression across a population of micro-reactors. Okano et al. (2014) showed, for example, that for a population of microwells containing the same number of copies of DNA, expressed in extra-cellular media of the same concentrations, the rate of the synthesis of a protein associated with a high-order kinetics presents an optimal volume in the range of 56–350 fL [30]. This emphasizes the competition between two timescales: The diffusion of macromolecules across the reactor and their condensation at higher concentration. Furthermore, boundary conditions may also have an activating or inhibiting effect on metabolic functions [16,31].

Owing to the high surface to volume ratio of tiny cells on the order of few microns to 100 µm, the physical boundary is not just a solid wall, but plays significant roles for intracellular biochemical reactions. Recently, the regulatory effect of the compartment is increasingly recognized and characterized in CFE systems. Sakamoto et al. (2018) performed CFE in emulsion droplets of various sizes [16] (Figure 2B). Droplet sizes from 10–100 µm in diameter show that the CFE levels scale to droplet radius with *R*^4^, in contrast to the scaling of *R*^3^ expected from the droplet volume. This result is reproduced by a simple mathematical model of repressing surfaces in which a higher surface to volume ratio leads to lower gene expression efficiency. Kato et al. (2012) showed that the surface reaction is activated using charged lipids [32]. Matsuura et al. (2012) showed that in the presence of a very low number of DNA copies, the gene expression level of a tetramer fluorescent protein shows size-dependence because the small compartment has an effectively higher concentration of monomers [33]. These studies show that the compartment itself has a significant influence on gene-expression, emphasizing the active regulatory roles of physical confinement.

#### 2.1.4. Kinetics and Noise in Cell-Free Gene Expression

Measuring the kinetics of protein synthesis in microwells is highly more efficient than in droplets, as microwells do not move because of the presence of a residual flow inside the chamber, they do not coalesce as they can never be in contact with one another, they do not spread onto the substrate, and do not suffer any gravitational sinking. In addition, the curvature of their interface is much less significant than for droplets, reducing the hindering of light intensity measurements.

**Figure 3:**
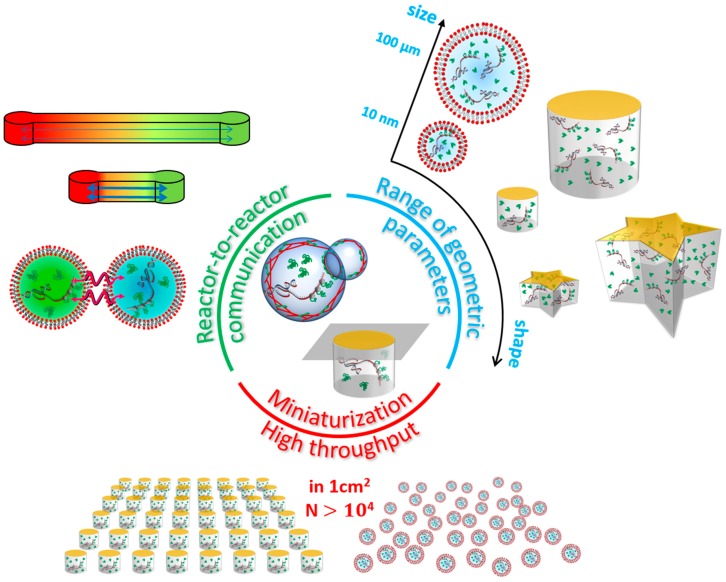
Artificial systems allow monitoring simultaneously very large populations of micro-reactors in very compact chips, with much less variability in the sample than in living systems. They also offer a significant flexibility in the control of the geometric parameters, as well as the interactions between individuals, without strongly perturbing the metabolism.

The measurement of the kinetics of gene expression reveals characteristic time scales involved in the degradation of mRNA and proteins. It also gives valuable information on the dominant steps that regulate the production rate of a given protein. That measurement was made possible thanks to the simultaneous screening of a large number of microwells with designed geometry. The burst-driven nature of the protein synthesis has given rise to numerous studies emphasizing the universal features of gene expression in living microorganisms [34,35,36,37]. Microwells can be used to elucidate those features (Figure 3).

A clonal population, sharing the exact same genetic information, eventually displays variability in its observed metabolic properties, such as protein concentrations. Numerous studies using actual bacteria displayed such variability [38,39]. For this study, two approaches are possible: On the one hand, one can study the natural system, culture bacteria, select a clonal population, and count the bacteria thanks to specific markers or reporters. As living cells remain complex systems, it is usually challenging to find the specific causes of the variability. The classical example is that Elowitz et al. designed two-kinds of genes, RFP (red fluorescent protein) and GFP (green fluorescent protein), is encoded in single DNA sequence inside bacteria *Escherichia coli* [40]. Depending on the environmental condition, such as temperature and the amount of nutrients, gene expression levels of RFP and GFP varies bacteria to bacteria. Consequently, bacterial color changes from red to green, which is the direct observation of gene expression noise (external noise). At the same time, the gene-expression level also varies, due to the inherent heterogeneity of the DNA copy number and stochastic nature of the gene expression reactions, which creates other kinds of noise (internal noise). Thus, these noises may affect the amount of protein expression variability, resulting in cell-cell variability for the developmental process where initial small variations are amplified by feedback mechanisms and eventually guide cell fates [40].

Several experiments attempted to find out what regulates that variability. In a bacterial population, such a measurement is made difficult as cellular division and mutation may hinder the screening. On the other hand, one can create a model system that contains minimal ingredients to mimic simple living organisms. In microwells and liposomes, once a micro-reactor reaches its equilibrium state, its chemical composition remains constant.

To see the global trend of the stochastic nature of the noise, we need a population level analysis. Thousands of liposomes can be filled at once, each of them with different geometric properties. Nishimura et al. (2015) showed that the fluctuations of the number of proteins synthesized, for two different proteins in the same cell-sized compartment, are correlated, whereas that correlation vanishes if the encapsulation is performed post-reaction [41]. The correlations observed in cell-sized compartments are also similar to those found in actual bacterial populations (Figure 3, bottom).

On the other hand, Karig et al. measured the gene expression fluctuation over time in 20 fL microwells made of PDMS [21,42] (Figure 2D). They showed that the magnitude of the gene expression noise is distributed along a non-Poissonian probability distribution function, with a Fano factor larger than 1 and could conclude that the dominant source of intrinsic noise in the gene expression lied in the translational burst during which several protein molecules are synthesized from the same mRNA molecule. Similarly, to those results, Salman et al. (2012) showed that the gene expression in a population of identical microwells also presents a distribution of yields stretched to the right [43]. This underlined the multiplicative nature of the noise of each of the reactions in the gene expression cascade.

### 2.2. On-Chip to Address Limits of Liposomes and Droplets

Gene expression in droplets offers a lot of flexibility in the handling of micro-reactors. It is easy to vary their composition, sort them and store them at given locations. However, a limitation shared by droplets, vesicles and liposomes micro-reactors is their shape: Due to the dominance of capillary forces, it can hardly be non-spherical. Furthermore, the size distribution of the produced micro-reactors remains broad and it remains challenging to accurately control its width. Those fluidic reactors can also aggregate and eventually coalesce.

Those limitations do not exist in the case of rigid compartments, namely, microwells. Those can be carved with a wide variety of sizes, shapes and suitable placements. Depending on the technology used, their volume can be as low as tens or hundreds of attoliters [44]. Although microwells are traditionally used as cylindrical micro-reactors, any other shape is also allowed. With the recent photolithography techniques, it is even possible to fabricate polygonal shapes with sharp corners [45] (Figure 3, right). Microwells also offer a much better control of the transport of matter as most of the substrate used, e.g., SU-8 photo-resist, are impermeable to water. This is a very easy way to ensure the complete insulation of the reactive media within a confined space. Transfer of chemical material can also be directed specifically along microchannels, and flows can be sequentially driven in order to obtain specific mixing conditions [46,47]. These allow to accurately and dynamically control the experimental conditions of biochemical reactions under confinement.

The choice of fabrication material is critical when it comes to designing an experiment in a microfluidic chip. Each material has its pros and cons; thus, the fabrication method and materials must be chosen accordingly to the specifications of the experimental system. The vast majority of the materials used falls into three categories: Inorganic materials, polymers and hydrogels [48]. When dealing with living cells and lab-made biological systems, in addition to the ease and cost of fabrication, two criteria come forward: On the one hand, the biocompatibility of the fabrication material [49], on the other hand, the permeability to gas, O_2_ in particular. We discuss inorganic materials and polymers: Those are the most widely used fabrication materials for microfluidics in biology.

*Inorganic materials,* such as glass and silica, offer a very high insulation, as well as durability, as they are impermeable to gas, chemically resistant to most solvents and very stiff. However, they are expensive materials, require expensive equipment to be fabricated and sealed, and cannot be used for long-term biological use. Their charged surfaces also increase the likelihood for proteins to adsorb, leading to cell adhesion and lower biocompatibility.

*Polymers* are the most popular fabrication materials. They come in a wide range of physical-chemical properties, are cheap, require inexpensive equipment and can be carved with a resolution down to 100 nm. Among polymers, elastomers, such as polydimethylsiloxane (PDMS) are the most commonly used. They are easy to bond to other materials, such as glass, simply with on oxidizing plasma activation. They owe their popularity to their bio-compatibility and their high gas permeability that makes them the fittest material for cellular culture. However, that advantage also comes with a fair number of drawbacks: PDMS offers very low resistance to organic solvents, can adsorb biologically, as well as hydrophobic molecules, at its surface, and is prone to water evaporation. In addition, PDMS is very difficult to functionalize. Nonetheless, its ease of fabrication and versatility make it the most used fabrication material in prototyping microfluidics.

In addition to elastomers, thermoset polymers, such as SU-8 are used for their higher mechanical and chemical resistance, as well as their impermeability. Their high strength allows them to be used in the fabrication of true 3D circuits as well high aspect ratio and free-standing structures. Their biocompatibility is ensured by the low adhesion of cells at their surfaces. However, their rather high cost and their limited bonding techniques reduce their use to mold fabrication, rather than micro-fabrication material.

Finally, thermoplastic materials, such as polystyrene, polyethylene and fluorinated ethylenepropylene are rather used for commercial scale fabrication. Their sealing is uneasy and requires either high temperatures or glues, yet they offer more versatility and durability in terms of surface coating or grafting than PDMS, along with a higher chemical resistance. Their low gas permeability limits their use in biological systems.

#### 2.2.1. Biological Membranes on a Chip

Compartmentalization is an important feature of living systems, and the nature of the boundary of the cells is crucial to ensure the stability and functionality of that compartmentalization. Some studies aim specifically at the functionalization of biological membranes, such as phospho-lipid bilayers, regardless if they enclose a bio-reactor or not. Although it is not easy to generate liposomes of a given shape and size, several techniques have been developed to generate planar membranes of given size and shape. Phospholipid bilayers can be set at the bottom of microwells [50,51] and form a large array of membrane patches. This allows studying the adsorption of proteins and drugs at biological membranes, but without any transport across them. It is also possible to study specifically trans-membrane transport by forming an array of microwells sealed by a biological membrane. This was achieved using similar methods, but in different geometries [44,52]. This opens the way to the functionalization of biological membranes with controlled geometric properties.

#### 2.2.2. Communication between Reactors

The richness of the behavior of microorganisms is revealed in the light of collective properties. This is made possible by their abilities to communicate with one another and with their environment. Various mechanisms have been developed by nature to allow bacteria to sense the presence of nutrients or pollutants in their vicinity, or communicate with neighboring microorganisms, either to prey on them or to flee. This requires the membrane of those microorganisms to be functionalized and let the medium it encloses interact with its outer environment. As an example of cell-to-cell communication, spatiotemporal patterns have been observed in engineered bacterial colonies with sender and receiver cells [22]. By setting the desired environment for the micro-bio-reactors, microfluidics can bring evidence of the cell-to-cell communication mechanisms at play in simplified systems. Not only the communication between cells has been observed, but also the communication between droplets and actual cells has also been reported [53], which enables the communication between the artificial realm and the natural world.

One of the important features of communication is the position-dependent gene expression gradient, which is a key process in morphogenesis. To quantitatively characterize such a protein diffusion-mediated communication between cells, Schwarz-Schilling et al. (2016) established a one-dimensional array of communicating water-in-droplets within microchannel [53]. On the other hand, to investigate the multi-cellular communication, Niederholtmeyer et al. (2018) developed cell-mimics covered with porous polymer membrane capable of gene expression and cell-cell communications mediated by diffusive protein signals using a microfluidic technology [54]. In the cell-sized porous compartment, DNA-hydrogel is confined, thus only expressed proteins diffuse across the compartments. By employing the cell-free expression system as the outer solution, this porous membrane allows cell-mimics to exchange inner solution and express genetic codes. This cell-mimics can communicate via diffusive protein signals, activating the neighboring cells, and collectively responses. They encapsulated this cell-mimic in 4.5 µL of the cell-free reaction solution, titrated the number of cell-mimics and measured the fluorescent intensity of each cell-mimics. Remarkably, they found a sharp transition from gene expression ‘off’ to ‘on’ state as the number of cell-mimics in a droplet increase, which resembles bacterial quorum sensing detecting cell population with the concentration of expressed molecules.

#### 2.2.3. On-Chip Communication

Although collective properties have been observed, it is usually difficult to ensure a homogeneous living sample. The use of an artificial system reduces the sources of variability and helps focus on the significant mechanisms at play in multi-cellular communication. However, the connectivity between droplets or liposomes, the distance between them, and the nature of their interaction are yet very difficult to set.

Microwells, however, do not suffer from those limitations. One can easily design a network of microwells with a specific connectivity in the form of micro-channels (Figure 3, left). Information between micro-reactors is exchanged in the form of the diffusive transport of chemical species. That signaling can mutually affect the gene expression taking place in the microwells and give rise to feedback loops. It is then possible to have biochemical reactors that generate pulses and oscillations. In particular, Karzbrun et al. (2014) demonstrated spatiotemporal patterns can emerge in microwells connected by microchannels [55] (Figure 2E). The coupling is achieved through diffusion of molecular species; a reaction-diffusion system is then set. Reactors can even synchronize their oscillations [56]. So far, at the quantitative level, various kinds of metabolism and mechanics orchestrated by gene expression and cell-cell communications have been characterized in in vitro reconstitutions. Therefore, microfluidic technologies will bring a deep understanding of biological functions from single cell level to the multi-cellular level.

Table 1 summarizes the main geometries used for cell-free bioreactors along with their characteristic dimensions. Each geometry offers its advantages and drawbacks, therefore, there is no ideal geometry for general biological use. They are rather complementary, as microwells and microchannels are more robust and versatile but also more artificial systems, while droplets and liposomes offer less versatile but closer to actual living systems.

## 3. Compartmentalized Active Dynamics

In the first part shown above, for the sake of the understanding of the design principle of artificial cells, it is discussed how compartmentalization can affect the property of biological systems in terms of chemical effect. The ratio of surface area to volume defines the scaling behavior of gene expression as discussed above, while the physical effect of a compartment is not limited to its size alone. Indeed, the cellular boundary has geometric shapes, such as a rod (*E. coli* bacteria), a sphere (micrococcus bacteria) and this boundary condition is essential to reliably make division plane for symmetric cell division. MinD and MinE proteins are involved in bacterial cell division by finding mid-point between two poles of rod-shaped bacteria [57]. These proteins constitute reaction-diffusion system on the surface membrane and self-organized surface waves emerge on supported lipid bilayer [58,59,60]. It has been known that nonlinear self-organized pattern strongly depends on boundary conditions in general. The confined MinD-MinE system can sense the curvature of boundary condition and its surface planar wave can be controlled by the global shape of chemical containers [61]. A similar approach has been recently applied to the FtsZ, bacterial homologue of tubulin and its spontaneous localization does not depend on the shape of boundary [62], suggesting that this protein can robustly localize and proceed to cell division at any local curvature of confinement. Thus, microfluidics offers great potential to pursue the relationship between biological systems and the geometric shape of compartments. How do cells sense their geometry and how do cells control their self-organization through physical interaction with the boundary wall? To answer these questions, we next focus not only on chemical species, but also on biophysical self-organization of motile elements, which are called active matter.

### 3.1. Collective Motion

#### 3.1.1. Collective Motion of Active Biological Fluids

Active matter draws its energy from the conversion of chemical reactions into mechanical work. It is ubiquitous across size scales, from microscopic motor proteins, mesoscopic microorganisms, to macroscopic individuals like us. Assembly of such motile elements is well-known to exhibit a variety of collective behaviors, such as flocking and vortical motion. Not only does compartmentalization play an important role at the single cell level, but also for active matter collectively moving through mechanical interactions in population level. Recent studies have found that geometric boundaries can control ordered phases of active matter from cytoskeleton, bacteria and synthetic colloids to locust owing to well-controlled experimental systems [63,64,65]. Here we present the importance of geometrically controlling the collective behavior of a large number of self-propelled microswimmers along with the recent development of microfabrication techniques that allowed that control.

**Figure 4:**
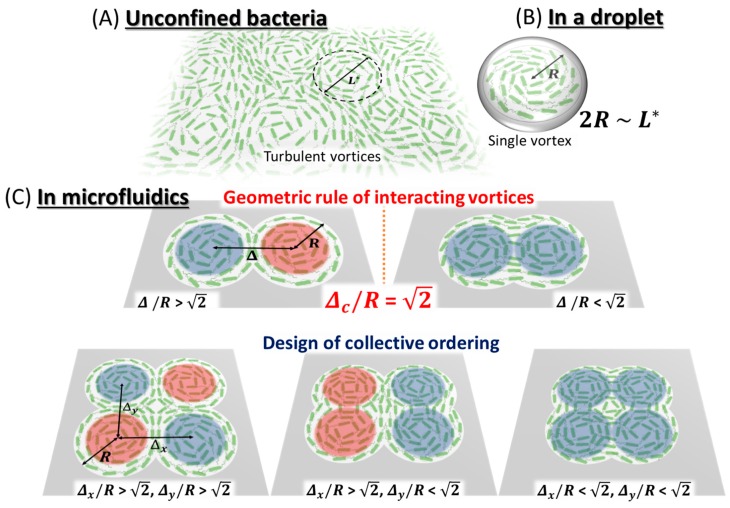
Schematic of the self-organization in bacterial suspensions from turbulent to rich ordered structures. (**a**) Meso-scale turbulence in a dense suspension of bacteria confined in the quasi-two-dimensional plane, which contains bacterial vortices having a characteristic size L*. (**b**) Bacterial suspension confined in a droplet. It can be stabilized into a single vortex by the confinement of a circular boundary having radius *R* comparable to L*/2. (**c**) Controlling collective ordering of bacteria by designed microwells. Doublet circles induce two distinct spinning patterns ruled by *Δ*/*R*=$\sqrt{2}$ (defined by the ratio distance between two circles *Δ* and radius *R*). The geometric rule allows us to control rich patterns [73].

For living active matter, rod-shaped bacteria have been extensively studied in both experimental and theoretical studies. In a quasi-two-dimensional plane, dense bacterial suspensions form swarming behavior, where disordered motion with transient ballistic motion (jets) appears from random bacterial swimming. However, pioneering work by Wu and Libchaber (2000) showed that this disordered state in a dense bacterial suspension is not just chaotic motion, but rather transiently ordered motion of vortex like swarming could be present [66]. They used silica beads tracer particles of few microns in a quasi-two-dimensional bacterial bath, and analyzed their Brownian trajectories. Indeed, the suspended particles are stirred by bacterial swarming and therefore the mean square displacement (MSD), or velocity correlation function of tracer beads can report the transient ordered state of bacterial collective motion although long-term behavior is regarded as random motion. MSD shows a rapid increase at shorter time-scale as *t*^2^, whereas the slope of MSD becomes proportional to *t* at longer time-scale. The cross-over at the characteristic time indicates ordered collective motion with coherent heading angle is emerged from apparently chaotic motion of swarming bacteria. Afterwards, many researchers have studied unconfined dense suspensions of swimming bacteria which exhibit turbulent-like collective behavior consisting of transient vortices and jets [67,68,69,70] (Figure 4a). In order to elucidate the difference between features of self-sustained turbulent state in microbial suspensions and those of classical turbulence in passive fluids, Wensink et al. (2012) evaluated velocity correlations and flow spectra calculated from the flow field of bacteria. Unlike the energy spectrum of classical turbulence, that of bacterial turbulence showed a characteristic peak, suggesting that the emergent vortices have a certain typical size. Moreover, they reproduced such property of collective bacterial behavior by means of simulations of a minimal 2D self-propelled rod (SPR) model, implying that collective bacterial dynamics is dominated by short-range interactions.

#### 3.1.2. Geometric Principle of Active Bacterial Vortices

How can one find ordered structure behind seemingly disordered motion in the group of bacteria? Studies for controlling patterns have attracted considerable interest, due to its potential in exploiting the underlying mechanism as a universal feature. A simple, but promising, means is to give a boundary condition whose geometric size is comparable to the coherent (characteristic) length of the internal ordered structure. Wioland et al. (2013) demonstrated this approach to isolate single bacterial vortex of *Bacillus subtilis* by putting the bacterial suspension in a water-in-oil droplet [71]. When *Bacillus* bacteria are confined in a droplet whose diameter is smaller than the characteristic length scale of meso-scale turbulence in free boundary, the group of bacteria starts to align the heading angle and then form coherent rotational mode as vortex-like structure (Figure 4b). Because the bacteria itself is rod-shaped, steric interaction between the boundary of the droplet and individual bacteria occurs, consequently aligns the direction of motion as a stabilized vortex. Indeed, the flagella rotation, namely propelling helical tail, pushes surrounding fluids at rest against the heading angle, which means bacteria also have hydrodynamic interaction with other bacteria or boundary wall. The boundary of the droplet is water-in-oil surface behaving like a solid wall, because of the viscosity difference between the inner and outer medium, the interface can be considered as a solid wall. This shear stress acts on bacteria swimming close to the boundary, and then turns their direction of motion opposite to the vortex rotation inside. Thus, by putting boundary condition onto the dense suspension of bacteria, the emergent dynamics and structure of vortex are stabilized, thanks to physical interaction with boundary.

The next question is how individual bacterial vortices interact with each other in a quasi-two-dimensional space. Using microfluidic devices made of PDMS, Wioland et al. (2016) extended circular geometries to lattices of circular cavities containing a dense bacterial suspension and connected by channels with width *w* [72]. In such chambers, two distinct ordered phases were observed: The ferromagnetic order of bacterial vortices rotating in the identical direction and the anti-ferromagnetic one of bacterial vortices rotating in opposite directions. The transition between these patterns is governed by the width of the channel *w*. Their experimental results exhibited fundamental similarities with quantum systems, suggesting the potential to capture collective behavior in active systems by using the theoretical concept to describe magnetism.

In spite of the suggestion that the rotation of bacterial vortices is affected by geometric constraint, it has not been clear what types of geometric rules are present behind the transition of collective bacterial ordering. To answer this question, Beppu et al. (2017) developed a microfluidic device in order to study the collision rule of a pair of bacterial vortices [73] (Figure 4c). Understanding the mechanism by which collective motions of self-motile elements are organized into ordered patterns is a central subject in the emerging field of active matter physics. As for the dense bacterial suspension, in a doublet of circular microwells, two vortices emerge, but their spinning directions show two distinct phases from the parallel pattern (ferromagnetic vortices, FMV) to anti-parallel one (anti-ferromagnetic vortices, AFMV). Let us note that the size of colliding vortices was set close to the characteristic length L* of meso-scale turbulence present at the emergence of collective motion. The boundary of doublet circles can be expressed by two parameters, the size of circular microwell *R*, and the distance between two circles *Δ* (defined as 2*R*cosφ with the elevation angle of φ). The transition of vortex pairing from FMV to AFMV occurs around at *Δ*/*R* = 1.4 in the experiment. To account for the geometric transition of active vortex pairing, the author analyzed self-motile point particles based on a Vicsek-style model [74], with confinement of doublet circles. Mean-field approximation reveals that non-dimensional quantity *Δ*/*R* governs the transition as seen in experiments, and the analytical solution yields the transition point as *Δ*/(2*R*) = cos(π/4), that is *Δ*/*R =* $\sqrt{2}$ = 1.414…, at which FMV and AFMV occur at equal probabilities. The Vicsek-style model reflects the interaction between bacterial vortices prefer to align at same direction in order to minimize the change of heading direction, as ferromagnetic interaction of spins. This geometric rule also accounts for more complex patterns, including pure FMV and AFMV, and also the coexistence of FMV and AFMV [73]. Remarkably, the above geometric rule can explain such a variety of the emergent patterns of bacterial vortices, revealing a design principle for bacterial vortices pairing. 

Table 2 summarizes the geometry-driven collective motions in bacterial suspensions with the representative size of the compartment.

#### 3.1.3. Topological Defects and Fluid Streaming

Bacteria are known as self-propelled active matter with its own polarity, while living active matter is not limited to such polar particles. Self-motile elements having interaction without distinction between head and tail belong to active nematics, named after nematic liquid crystal. A representative example of active nematics is epithelial cells in the two-dimensional plane, such as MDCK cells and HeLa cells [75,76,77,78,79,80,81]. Although those epithelial cells have an actual polarity of the leading edge and trailing cell body, their direction is not stable, but rather shows large fluctuation similar to back and forth motion. Moreover, because each cell makes alignment through physical contact and collision against neighboring cells, the collisional rule involved belongs to nematic alignment. As for HeLa cells, what is remarkable about active nematics is the emergence of topological defects in their dense population onto a two-dimensional surface. In a two-dimensional plane, the net topological charge has conservation law: For instance, in a circular boundary condition, the cell population where two +1/2 topological defects present is the energetically most stable state (ground state). Duclos et al. (2017) took a time-lapsed acquisition of HeLa cell population inside a circular boundary pattern on a PEG-PLL grafted glass surface [79]. At the early phase of collective migration, a number of topological defects, both +1/2 and −1/2 defects are present [80,81,82]. A striking feature in active nematics is that topological defects can spontaneously move and annihilate after the collision of two opposite defects. Annihilated topological defects make clear alignment of a group of cells and eventually collapse into a ground state of two +1/2 defects, thanks to the circular geometric constraint.

These topological defects spontaneously move in a confined space and occasionally drive fluid streaming similarly to what can be observed in a cell. Inside cells, the cytoplasmic streaming—fluid flow within the cytoplasm—is generated by the cytoskeletal network mainly composed of polar filaments and molecular motors. Confinement of active microtubule and kinesin motors allows one to test how elongation of microtubule bundle can drive cytoplasmic flow in cells [83,84]. In the bulk extract, where an actin-depolymerizing agent and a dynein inhibitor are added, the emergence of turbulent-like complex flow is observed, which is visualized by the presence of granules. According to the calculation of the equal-time velocity auto-correlation function, the correlation length that corresponds to the size of vortex flow is comparable to the length of single microtubule bundle. It has been also found that complex flows are induced by the elongation of the bundles. These results suggest that hydrodynamic interactions between extensile microtubule bundles result in complex flows, which is a mechanism similar to that of bacterial vortex flows. Furthermore, confinement of the extract into droplets induces rotational flow that emerges in droplets of 100–700 µm in diameter, while the size of vortex flow in bulk extract is approximately 50 µm. In addition, the rotational flow in the droplets is sustained for over 10 to 100 times longer than in the bulk. It has then been hinted that in addition to hydrodynamic interactions, mechanical interactions between extensile microtubule bundles and the boundary induce such large-scale temporally stable cytoplasmic flow.

### 3.2. Application

#### 3.2.1. Microfluidics to Harness the Power of Biological Fluids

Finally, controlling collective motion using microfluidics is of significance not only for revealing the universal feature behind collective behaviors, but also for further development of mechanical micromachines. Sokolov et al. (2010) demonstrated that a ’bacterial bath’ can drive sub-millimeter gears decorated with asymmetric teeth [85]. Despite randomly moving or chaotic motion of bacteria, directional rotation of gears was observed in the regime of the emergence of bacterial collective motion, and its angular velocity was controlled by the amount of oxygen. Moreover, Thampi et al. (2016) have introduced microfluidics with no asymmetric structure driven by the turbulence of active nematics [86]. A lattice array of cylindrical rotors placed in meso-scale turbulence exhibited the persistent antiferromagnetic spin state, such as earlier bacterial vortex lattices. They indicated that there is a certain geometric characteristic to control such persistent unidirectional flow, depending on the characteristic length scale of the meso-scale turbulence. These experiments have demonstrated how accordingly designed microfluidics can allow us to extract mechanical work from collective behaviors in active systems.

As for bacteria, a circular boundary stabilizes disordered phase composed of turbulent vortices into a single vortex, and geometric constraints on interacting vortices generate rich ordered patterns. These results reveal the intrinsic property, such as the characteristic vortex structure and the short-range interaction in motile entities. As for microtubules driven by motor proteins, confinement induces stable cytoplasmic flow on the larger scale than the intrinsic vortex size in bulk cytoplasmic solution. Suitably designed boundaries can provide the way to control the disordered phase and ordered phase in active systems.

Elucidating the mechanism of collective behaviors will provide fundamental understandings for broad biological phenomena, since cytoplasmic streaming, biofilm formation and even collective migration of cancer cells are still not well understood. Furthermore, the understanding of a universal feature of collective behaviors and advanced microfabrication may lead to practical applications. Constructing a theoretical model for collectively moving active living matter or inanimate one like robots will give insights into the design principles of active micromachine which can produce efficient mechanical work from collective motion.

#### 3.2.2. Microfluidics for Self-Organization in Three-Dimensional Space

Next challenge is presumably to control collective behaviors by controlling three-dimensional geometry, e.g., depth of microchannel or shell-like confinement. For example, Wu et al. (2017) observed the transition from turbulent to coherent flow in three-dimensional confined microtubules driven by kinesin [63]. What kind of geometry of three-dimensional confinement has first priority to explore the underlying principle of active matters? Simplest, but effective, geometry is spherical shell because the conservation of topological charge is still valid for nematic fluids. Keber et al. (2013) enclosed active nematics in the inner surface of the shell-like liposome, and they found that topological defects showed oscillatory displacement along the equator of the surface [87]. Such topological effects behind the ordered phase of active matter are crucial for better understanding of dynamics and emergent structure in various biological systems. Moreover, surface wrinkling patterns are generated on the surface of swelling hydrogel under lateral confinement force [88]. The grooves in a wrinkled surface may induce periodic order in collective motion of active polar fluids (bacterial suspensions) or active nematics (cytoskeletons). Moreover, a wrinkling-like folded structure is present, for instance in the intestinal epithelia. The mechanical interplay between folded geometry and active nematics of epithelial cells are a future challenge across the border of cell biology and active biological fluids. Although the technique to control three-dimensional geometry is not fully established even in microfluidics, such exploration in three-dimensional confinement will bring novel insights in developmental embryogenesis where cells grow, divide, and collectively move on the steric surface.

## 4. Conclusions

**Figure 5:**
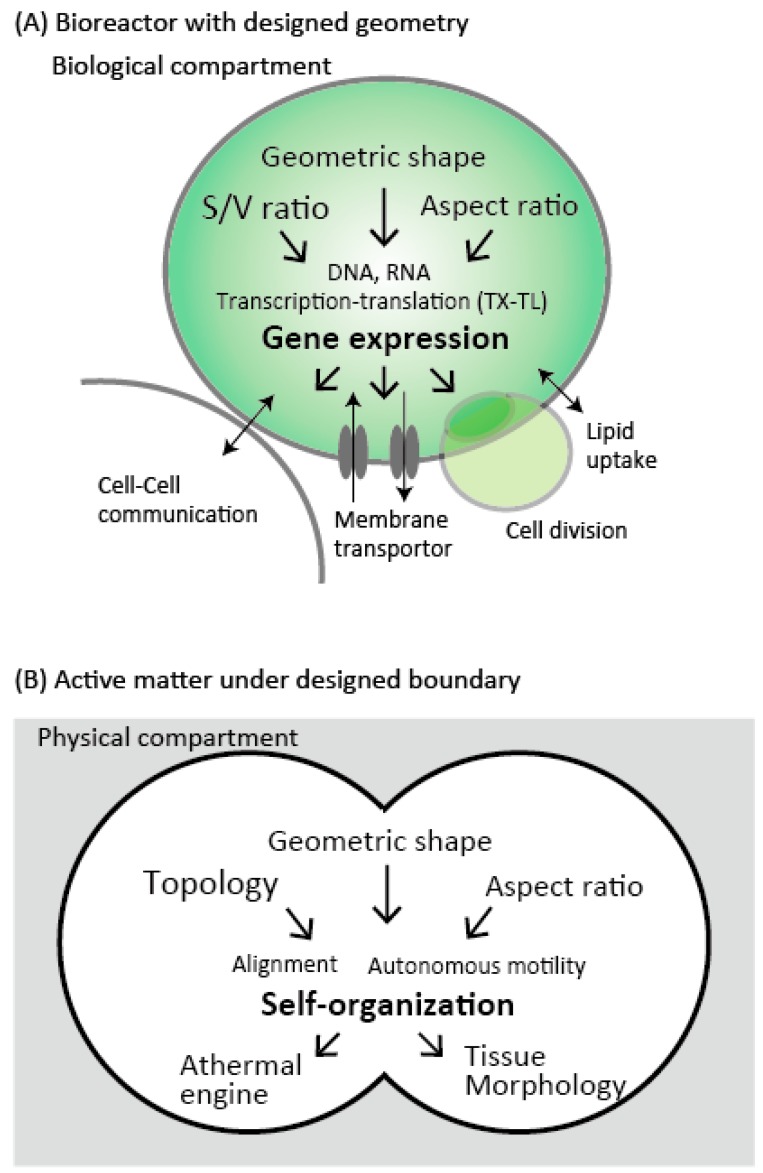
The effect of the geometric boundary for bioreactors and active matter. (**A**) Cell-sized confinement defined as biological compartment can be involved in biological function through geometry-induced TX-TL regulation. (**B**) The designed geometry of physical compartment is responsible for the control of self-organization of the group of active matter. The effect of geometric shape, its aspect ratio, and topology are essential to control self-organized patterns. The precise control of such pattern formation may be relevant to tissue morphogenesis or athermal engine.

In this review, we have described the microfluidic approaches for the study of synthetic biology and physics of biological fluids. Microfluidic technology facilitates precise control of biochemical species, such as DNA and proteins in a droplet, which is the important feature highly compatible with CFE. Quantitative experimental results further enable them to develop a simple mathematical model describing enhanced gene expression noise. Taken together, microfluidic technology is low-cost and offers versatile experimental platforms to investigate gene-expression reaction in droplets or microwells as live-cell mimics, enabling one to characterize the underlying mechanism of several biological processes (Figure 5A).

Furthermore, the designed geometric boundary is a key concept to control collective dynamics of active matters, from bacteria and cytoskeletons to eukaryotic cells (Figure 5B). By placing stem cell culture inside the circular boundary, the spatially ordered patterns of differentiated cells are reconstructed after growth and cell fate decisions [89,90]. Such tissue-on-a-chip technology requires microfabrication with designed geometry, and physical understanding of the interplay between biological matters and boundary shape is a fundamental question in order to develop a lab-on-a-chip method for synthetic biology or synthetic developmental biology [91]. While this application is mostly used in two-dimensional confinement, three-dimensional geometric confinement, e.g., spherical shell [92], will give new insight in mechanical aspects behind developmental morphogenesis in future.

## Figures and Tables

**Table 1:** Summary of cell-free TX-TL reactors. The geometric properties of cell-free reactors are classified with volume, characteristic length (radius or height), and shape. The density of reactors is also defined by the design of the microfluidic chip. Microwells and microchannels have large stability, but the construction of membrane-bound reactors is still challenging.

Reactor	Typical Volume Range	Smallest Geometric Parameter	Number of Copies per Experiment	Stability	Fabrication	Notable Properties
Test tube	10 µL to 1 mL	Radius: 1 mm	Up to 100	∞	-	Easiest
						Cheapest
						Bulk only
Droplet	10 aL to 1 µL	Radius: 100 nm	≥$10^{4}$	From hours to days	Simple	Very easy
						Requires two immiscible phases
						No functional membrane
					Well-controlled size	
						Only spherical
						Very limited cell-to-cell communication
Liposome	10 aL to 1 pL	Radius: 100 nm	≥$10^{4}$	A few hours	Difficult	Close to biological systems
						Functional membrane
					Wide dispersity of size	
						Lacks stability
						Only spherical
						Limited cell-to-cell communication
Microwell	10 aL to 1 µL	Height: 10 nm	≥$10^{4}$	∞	Simple	Easy
						Functional membrane
						Wide range of shapes and aspect ratios
					Well-controlled geometry	Boundaries not completely functionalizable
						Low stability of biological membrane
						No cell-to-cell communication
Microchannel	100 aL to 1 mL	Height: 10 nm	≥$10^{4}$	∞	Simple	Easy
						Wide range of shapes, aspect ratios and connectivities
					Well-controlled geometry	
						Best for cell-to-cell communication
						No functional membrane

**Table 2:** Summary of microwell geometry. The boundary conditions by which collective motion of active bacterial swimmers are controlled are boundary-free, cylinder, the array of the cylinder, and overlapped cylinder.

	Bacteria	Geometry	Height	Radius	Geometrical Control Parameter	Ordered Collective Motion	
Wensink et al. (2012) [70]	*Bacillus subtilis* (strain 168)	Boundary-free	5 µm	-	-	Meso-scale Turbulence (Typical Vortex Radius ~25 µm)	
Wioland et al. (2013) [71]	*Bacillus subtilis* (strain 168)	Cylinder	25 µm	35 µm	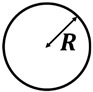 Radius *R*	Single Isolated Vortex	
Wioland et al. (2016) [72]	*Bacillus subtilis* (strain 168)	Array of Cylindrical Microwells	18 µm	25 µm	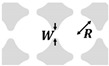 Channel Width *W*	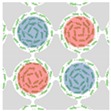 *W* < W* Anti-ferromagnetic Order	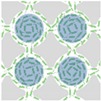 *W* > W* Ferromagnetic Order
Beppu et al. (2017) [73]	*Escherichia coli* (strain RP4979)	Multiplet of Cylindrical Microwells	20 µm	28 µm	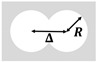 Radius *R* Distance Between Centers *Δ*	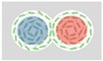 *Δ*/*R* > $\sqrt{2}$ Anti-ferromagnetic Vortices	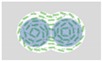 *Δ*/*R* < $\sqrt{2}$ Ferromagnetic Vortices
